# Modeling of Metal Powder Densification under Hot Isostatic Pressing

**DOI:** 10.3390/ma17081933

**Published:** 2024-04-22

**Authors:** Jingzhe Wang, Shesh Srivatsa, Zhanfang Wu, Zaiwang Huang

**Affiliations:** 1 State Key Laboratory of Powder Metallurgy, Central South University, Changsha 410083, China; wangjingzhe@csu.edu.cn; 2 Powder Metallurgy Research Institute, Central South University, Changsha 410083, China; 3 Srivatsa Consulting LLC, Cincinnati, OH 45249, USA; shesh.srivatsa@gmail.com; 4 China Iron and Steel Research Institute Group Co., Ltd., Beijing 100081, China; wuzhanfang@hipex.cn

**Keywords:** metal powder, hot isostatic pressing, consolidation, modeling, relative density

## Abstract

The consolidation of metal powders is a complex thermomechanical process, and the temperature has a significant effect on the density distribution in the compact. The consolidation process of metal powders with an average particle size of 10 μm, 25 μm, and 50 μm under hot isostatic pressure was simulated by finite element modeling. The distribution and evolution of the relative density after being hot isostatic pressing (HIP) under 1050 °C/130 MPa/4 h, 1150 °C/130 MPa/4 h, and 1250 °C/130 MPa/4 h conditions were simulated, respectively. The experimental data of HIP at 1050 °C/130 MPa/4 h were used to verify the modeling results via the geometric change in the container. The relative density difference between the simulated results and the experimental results at different positions was less than 2%. This methodology called “modeling prediction, experimental validation” can accelerate experimental discovery in an economic manner.

## 1. Introduction

Hot isostatic pressing (HIP) is a powerful process to densify metal powder into a desirable shape, microstructure, and properties. The relative density (RD) and distribution of an HIPed part are highly dependent on temperature [1,2], pressure [3,4,5], soaking time [6,7], and container geometry [8,9] since the densification process is primarily driven by elemental diffusion and the creep deformation of powders [10,11,12,13,14]. To densify it close to the theoretical density, in practice cases, a trial-and-error methodology is often carried out to obtain the optimized processing parameters and container geometry, which is highly costly and labor-intensive [15,16,17,18,19]. To address this, a new strategy of “simulation prediction, experimental validation” has demonstrated the advantage of accelerating the discovery of optimized parameters in an efficient and economic manner [20,21,22,23,24,25]. In this research, we provided a case showing the effect of temperature on relative density during HIP. Finite element modeling (FEM) was used to model the consolidation process of 316 L stainless steel powder under HIP at different temperatures, and experimentally measured data were used to validate the modeling results [26,27,28,29,30]. The simulation results clearly showed that the evolution of relative density is highly dependent on temperature. The geometry of the HIPed container was measured experimentally, and the modeling results were verified by density measurements at different locations.

## 2. Materials and Methods

The powder material was 316 L stainless steel and its composition is shown in Table 1. The powder was mixed with three groups of particles with a weight ratio of 1:1:1, and their average sizes were 10 μm, 25 μm, and 50 μm, respectively. After vibration, the initial packing density of powder was 4.9 g/cm^3^.

**Figure 1:**
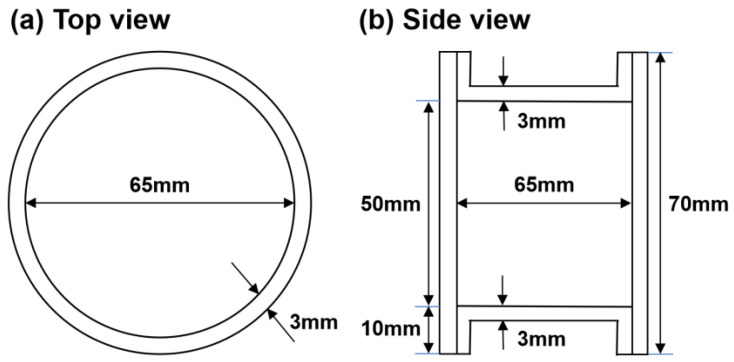
Geometry of cylindrical container for powder consolidation.

The container material was 45# low-carbon steel and its composition is shown in Table 2. The inner diameter, length, and wall thickness of the container were 65 mm, 70 mm, and 3 mm, respectively, as shown in Figure 1. The container was degassed at 550 °C and then sealed by welding under a vacuum of 1 × 10^−3^ Pa.

The hot isostatic pressure (HIP) device RD80, HIPEX, CISRI HIPEX TECHNOLOGY CO., LTD.,Beijing, China was used in the experiment. In the experiment, the specimen was slowly heated to the target temperature and then soaked for a certain time. At the same time, constant pressure was applied to the specimen. After the heat preservation, the specimen was cooled in the furnace. The experimental temperatures were 1050, 1150, and 1250 °C, employing a pressure of 130 MPa with a soaking time of 4 h.

## 3. Simulation Model

Based on the classical Von Mises theory, the basic function of the yield criterion for powder materials can be obtained, as shown in Equation (1).

(1) $$\sigma_{s}={\left[\alpha_{1}\left(3J_{2}^{\prime }\right)+\alpha_{2}{\left(J_{1}\right)}^{2}\right]}^{\frac{1}{2}}$$

where *α*_1_ and *α*_2_ are not a function of relative density, determined by the boundary conditions shown in Equation (2). *J*_1_ is the first invariant of stress, and $J_{2}^{\prime }$ is the second invariant of stress deviation. *σ_s_* is the yield stress of the powder.

(2) $$\left\{\begin{array}{c} {\left.\sigma_{s}\right|}_{\sigma_{2}=\sigma_{3}=0}=\sigma_{1}\\ {\left.\frac{d\varepsilon_{2}}{d\varepsilon_{1}}\right|}_{\sigma_{2}=\sigma_{3}=0}=v \end{array}\;\;\right.$$

where *v* is Poisson’s ratio and *ε* is strain.

From Equations (1) and (2):

(3) $$\left\{\begin{array}{c} \alpha_{1}=\frac{2}{3}\left(1-v\right)\\ \alpha_{2}=\frac{1}{3}\left(1+2v\right) \end{array}\;\;\;\right.$$

Substituting Equation (3) into Equation (1) yields the Kuhn yield criterion [31], as shown in Equation (4).

(4) $$\sigma_{s}={\left[3J_{2}^{\prime }-(1+2v)J_{2}\right]}^{\frac{1}{2}}\;$$

Since the yield criterion of the powder can be expressed by the surface of an ellipsoid in the principal stress space [32], the eccentricity e of the ellipsoid can be calculated by Equation (5). The eccentricity e determines the geometry of the yield criterion of the powder in the principal stress space.

(5) $$e=\sqrt{2\alpha_{2}/\alpha_{1}}$$

Based on the classical yield criterion of porous materials (Kuhn model), the commercial finite element software DEFORM V11.0 (Scientific Forming Technological Company, Columbus, OH, USA) was used for modeling. The steps used were listed as follows:

(1) The geometry of the powder and container was defined.
(2) The thermal property parameters and rheological curves calculated by JMatPro V10.0 software were used to define the material model.
(3) A finite element mesh was generated in the powder and in the container.

**Figure 2:**
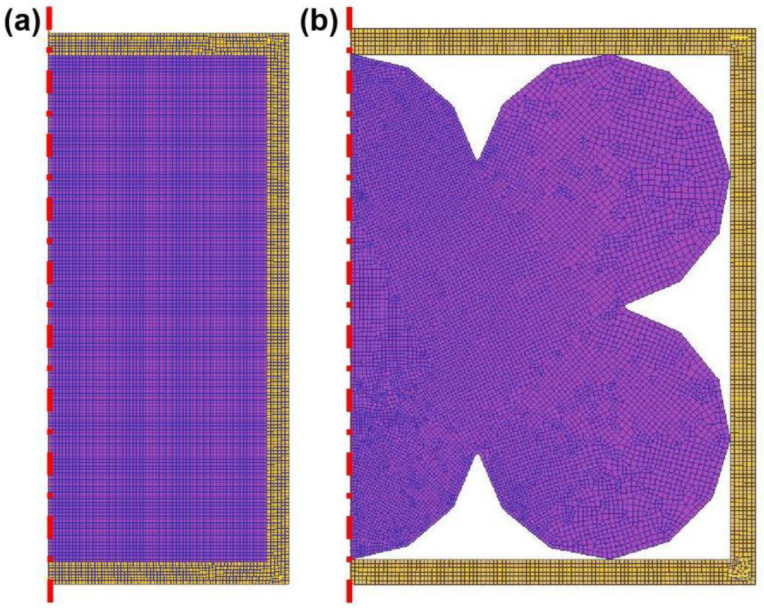
Finite element simulation of individual particles. (**a**) Macroscopic model meshing; (**b**) microscopic model meshing.

The meshing size was set to be 5000 (Figure 2). In order to investigate the microscopic deformation between powder particles, DEFORM finite element modeling was carried out to illustrate the evolution details. Assuming the symmetry of powder particles, a 2D geometric model and meshing was constructed, and the meshing size was 10,000 (Figure 2).

(1) The thermal and pressure boundary conditions on the outside surface of the container were specified. The temperatures were 1050 °C, 1150 °C, and 1250 °C, respectively. The pressure was 130 MPa and was loaded from outside to inside.
(2) The heat transfer and friction conditions between the powder and the container were specified. The thermal conductivity was assigned to be 11 W/(m·K), and the friction coefficient was 0.3.
(3) The simulation was started, to last for 4 h.
(4) When the shape change caused a degeneration of the finite element mesh, automatic re-meshing was performed to keep the mesh suitable for accurate calculations.
(5) The simulation was stopped at the end of 4 h (HIP time).
(6) The results were post-processed.

It should be noted that the modeling accuracy depended on the size of the finite element mesh. In this case, a total of 12,000 elements were used in the powder and container. This was adequate to provide an accurate numerical solution. Based on extensive prior experience, a larger number of elements will improve the accuracy only slightly (1–2%).

## 4. Results and Discussion

**Figure 3:**
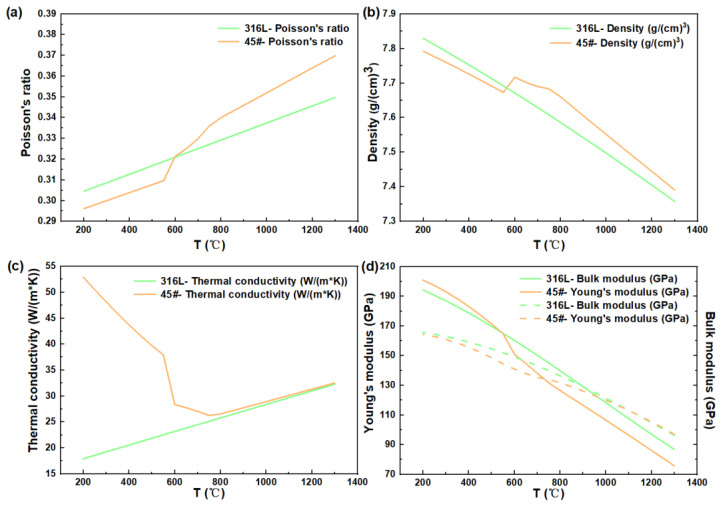
Thermophysical properties of 316 L stainless steel and 45# steel calculated by JMatPro software. (**a**) Poisson’s ratio, (**b**) Density, (**c**) Thermal conductivity, (**d**) Young’s modulus and Bulk modulus.

The thermophysical properties of 316 L stainless steel and low-carbon steel were calculated by JMatPro V10.0 software (Sente Software), as shown in Figure 3.

With the development of computational materials science, thermophysical properties can be accurately measured or estimated and are not a concern. These curves are used in DEFORM’s material modeling process, rather than using fixed values. This method can effectively improve the accuracy of the model.

**Figure 4:**
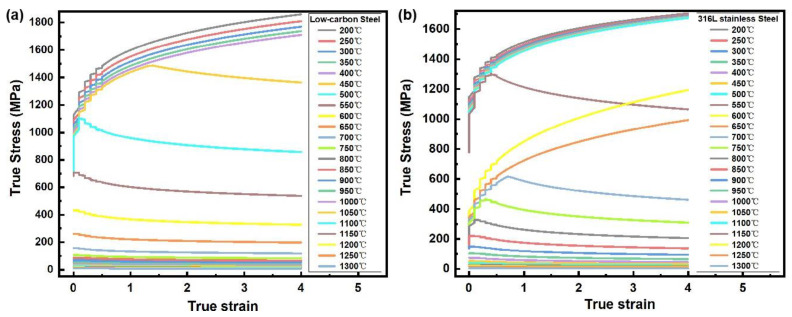
The flow stress data of powder and container materials at different temperatures calculated by JMatPro software. (**a**) Low-carbon steel (container); (**b**) 316 L stainless steel (powder).

The flow stresses of the powder and container material are regarded as the most critical modeling data in the process of DEFORM modeling. Here, properties calculated by JMatPro at different temperatures were used (Figure 4). It is good practice to validate the JMatPro predictions with selected compression tests and adjust the modeling parameters accordingly. The only boundary condition of importance is the pressure boundary condition, which can also be accurately controlled in a pressurized furnace.

The results showed that the peak stress of low-carbon steel and 316 L stainless steel decreased with the increase in temperature. When the temperature was less than 450 °C, the true stress of the low-carbon steel continued to increase with the increase in true strain below 4%. When the temperature was between 450 °C and 750 °C, the true stress of the low-carbon steel rapidly reached the peak stress and then gradually decreased to the steady stress with the increase in the true strain. When the temperature was higher than 750 °C, the true stress of the low-carbon steel remained steady. For the 316 L stainless steel, when the temperature was less than 700 °C, the true stress constantly increased with temperature. Among them, representative rheological curves were obtained at 550 °C and 700–1100 °C, that is, with the increase in true strain, the true stress rapidly reached the peak stress and then gradually decreased to be steady. When the temperature was greater than 1100 °C, the true stress of the 316 L stainless steel was nearly constant during compression. Combined with the thermophysical properties of the materials shown in Figure 3, it can be seen that the turning point of the thermo-physical properties of the two materials was near 600 °C, which accounts for the change in flow stress.

**Figure 5:**
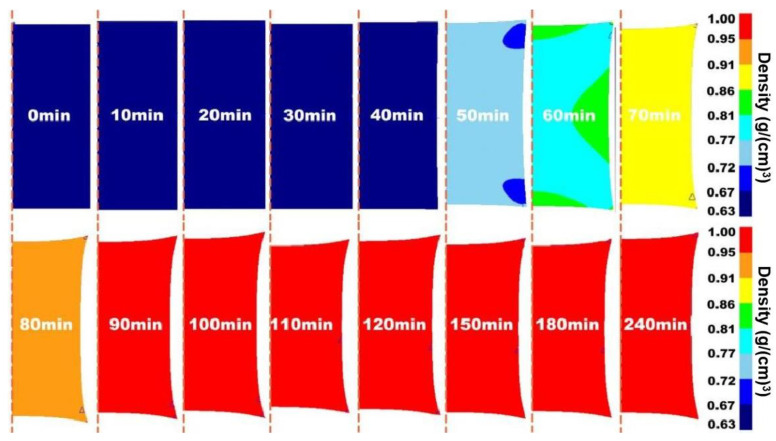
Two-dimensional visualization of densification process of 316 L stainless steel powder under HIP at 1050 °C/130 MPa/4 h.

**Figure 6:**
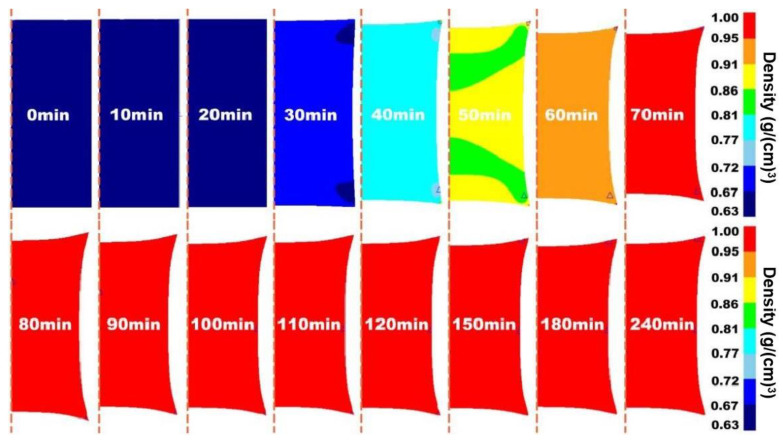
Two-dimensional visualization of densification process of 316 L stainless steel powder under HIP at 1150 °C/130 MPa/4 h.

**Figure 7:**
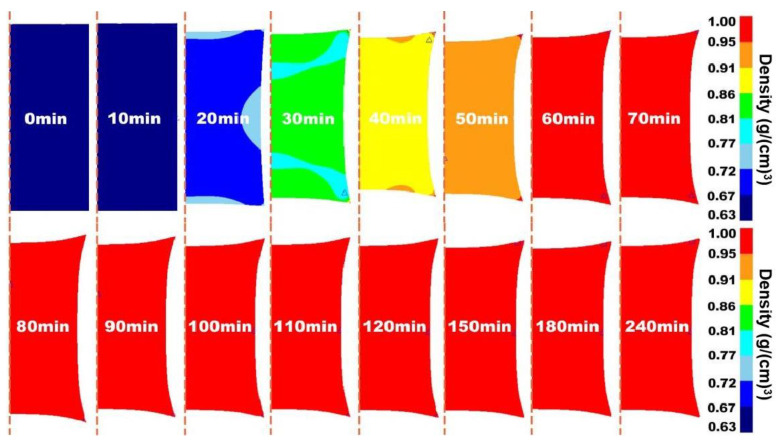
2D visualization of densification process of 316 L stainless steel powder under HIP at 1250 °C/130 MPa/4 h.

Prior to the experiment, the hot isostatic pressing (HIP) process of 316 L stainless steel was simulated by DEFORM finite element software to predict the density of the alloy, which provided the basis for the selection of conditions for the subsequent experiment. The simulation results are shown in Figure 5, Figure 6 and Figure 7.

Figure 5, Figure 6 and Figure 7 show the two-dimensional finite element simulation results of the HIP densification process of 316 L stainless steel powder at 1050 °C to 1250 °C when the pressure was set to 130 MPa and the pressure holding time was 4 h. For comparison, it can be clearly seen that under the same pressure and holding time, increasing the HIP temperature could effectively accelerate the densification process of the powder. When the temperature was 1050 °C, the powder needed at least 90 min to reach a relative density of 90%. With the temperature increasing to 1150 °C, it took around 70 min to achieve a relative density greater than 90%. When the temperature became 1250 °C, the time decreased to less than 60 min to reach that density. After an initially rapid densification, the relative density slowly increased with soaking time.

The results showed that the density distribution inside the powder was position-dependent and there was an irregular density gradient from the edge to the center before the specimen was fully densified. This is because an isotropic external load is applied in the HIP compression process, but the consolidation of the powder is not uniform in the actual experiment. The temperature gradient during densification leads to the anisotropy of powder configuration [33,34]. At the same time, a high temperature will cause the grain size of the material to increase and reduce the mechanical properties of the material. The deformation resistance at a low relative density is lower than in other regions. The heterogeneity of deformation resistance within the specimen leads to the difference in densification rates in different regions, which was also indicated by the density gradient in the model.

**Figure 8:**
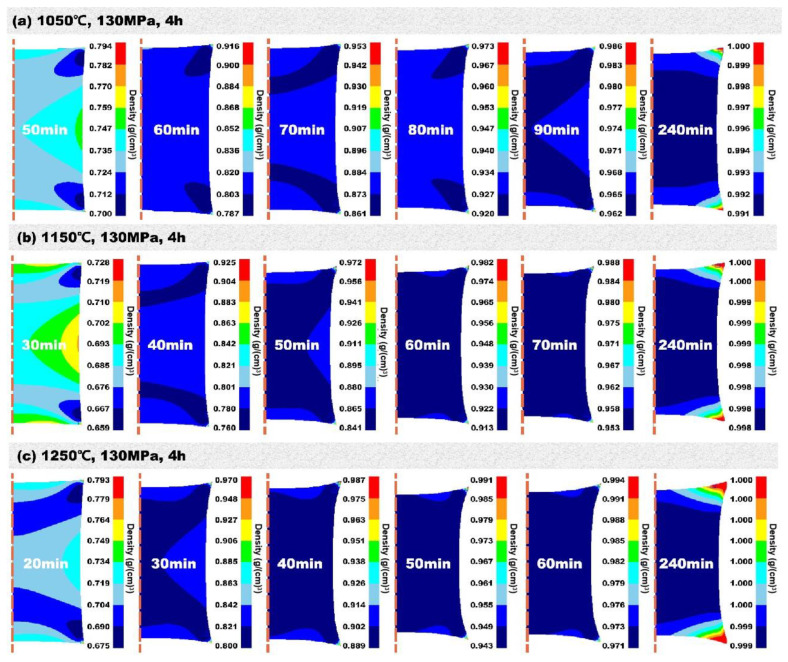
Relationship between densification degree of 316 L stainless steel and hot isostatic pressing temperature and holding time.

To more clearly compare the change in density inside the specimen, the density scale of the simulation results was changed. Figure 8 shows the simulation results of the relative density under different temperatures and soaking times when the pressure was fixed to be 130 MPa. At 1050 °C (Figure 8a), a general trend was that the relative density increased with increasing soaking time at any position of the billet. In the initial stage (i.e., 50 min), the powder inside the billet had a higher relative density than other positions, except at the edge corner due to the container effect, since metal powder can be regarded as a porous solid. The densification was further developed with increasing soaking time until 240 min, and the relative density in most parts of billet reached more than 99.1%. Similarly, under 1150 °C (Figure 8b) and 1250 °C (Figure 8c), the densification followed similar behavior and a higher temperature corresponded to a higher densification rate. After 240 min, the relative density throughout the billet generally increased with increasing temperature when compared with Figure 8a–c.

During HIP, the densification rate in the center of the specimen is faster than that at the edge of the specimen. However, after holding for a certain period of time, the specimen will eventually be densified. In the simulation process, the temperature distribution in the hot isostatic pressing process is uniform. The difference in the densification rate at different locations due to uneven temperature distribution is not considered. The rapid densification in the center of the specimen is due to the fact that in the process of hot isostatic pressing, the pressure is usually transferred from the outside to the inside. Since the forces inside the specimen are more complex and there is stress accumulation in different directions, the densification speed inside the specimen is faster when the accumulated stress is greater than the outside of the specimen. The densification of metal powder compacts under hot isostatic pressing and high temperature is essentially a multi-scale process problem, and the macro-scale distortion is caused by the interaction of local micro-scale powder particles.

**Figure 9:**
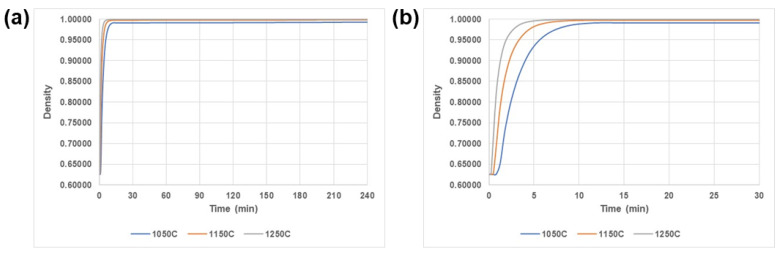
The dependence of relative density of 316 L stainless steel powder on temperature and soaking time when the pressure is fixed to be 130 MPa. (**a**) 0–240 min; (**b**) 0–30 min enlarged image.

Figure 9 shows the evolution of the relative density at the central position of the billet under different temperatures and soaking times when the pressure was fixed to be 130 MPa. A typical HIP cycle consists of three stages: elevating pressure and temperature to peaks, then holding them for some time, and finally decreasing them to normal states. Pressure and temperature are transmitted to the powder through its container. The highest holding temperature is usually greater than 0.7 times the melting point. At the beginning of HIP, the specimen rapidly densifies and a higher temperature corresponds to a higher densification rate. The densification rate is dramatically improved by increasing the pressure applied. This can be attributed only to the pressure that causes the particles to flow, and to the motion of the particles eliminating porosity. After a critical value, the densification rate becomes stable. At this point, the applied pressure reaches its peak, and the plastic strain and relative density also reach the holding stage. At the same time, the equivalent elasto-plasticity performs the typical characteristic phase of transient creep by rapidly decreasing the strain rate due to work hardening. The mechanism of densification at this stage is attributed to the following effects: elasto-plastic deformation, particle rearrangement, and collapse. The relative motion of isolated pores with particles involves relative translation and rigid body rotation. These curves indicate that 20 min is enough to reach a relative density of 240 min, as shown in Figure 9b, which is important to save processing time and costs in industrial practice.

**Figure 10:**
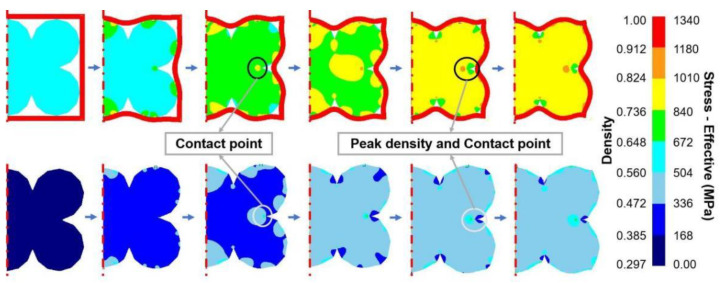
Density and stress changes in powder particles during continuous densification in HIP process.

Figure 10 shows the evolution of the relative density of powder particles during the densification process of HIP. The temperature is 1150 ° C, the pressure is 130 MPa, and the end time is 40 min. The top row is the density change, and the bottom row is the effective stress change. It can be seen that the density of the edges of the powder particles in contact with each other increased first, and the density inside the powder particles increased uniformly with the increase in HIP time. The uncontacted parts of the particles remained low in density until the contact density increased by leaps and bounds. This was because the interior of the two particles was in a uniformly dense state at this time, and the state of the contact position was loose. At the initial stage of contact, the contact area is subjected to force between particles as well as pressure by the HIP. Therefore, the relative density in the parts between the particles will continuously increase after contacting. This can be verified by comparing the stress changes in powder particles during the densification process of HIP. The relative density of the powder is positively dependent on the effective stress inside the powder particle. When these powder particles are taken as a whole specimen, the stress inside them is initially homogeneous, causing a rapid increase in density in the central part, which then gradually radiates towards the outside of the specimen.

This also explains why the density of the center of the specimen was greater than that of the edge in the simulation. When the stress concentration is established in the center of the specimen, it will cause the particles in the center of the specimen to first deform and rearrange. When the center of the specimen is dense, it has a stronger ability to resist constant external pressure. At this time, the specimens near the edge are still not dense, and this stress concertation gradually radiates towards outside. The simulation results clearly demonstrated the evolution process of relative densification during HIP, which was inaccessible during the experiment.

**Figure 11:**
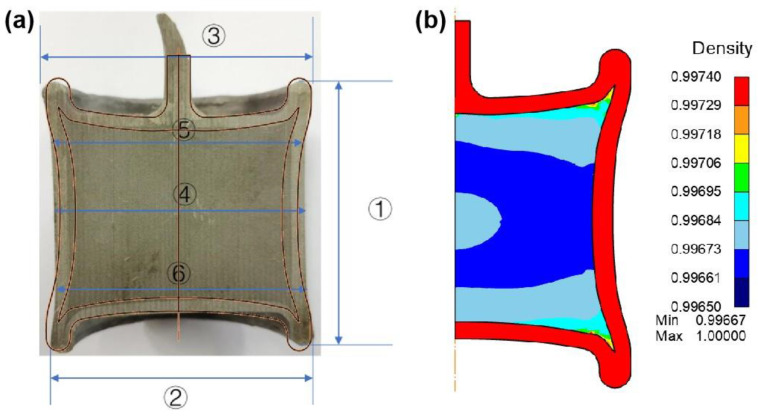
Hot isostatic pressing (HIP) experiment and DEFORM finite element simulation results of 316 L stainless steel. (**a**) Schematic diagram of the size partition of the HIP capsule; (**b**) DEFORM finite element 2D simulation results.

To validate the modeling results, the size of the HIPed container under 1150 °C/130 MPa/4 h was measured and used to compare with the numerically simulated ones. In order to better compare the results of the experiment and numerical simulation, the modeling process adopted the exact shape and size of the experiment. Figure 11 demonstrates the experimental and modeling results of the container configuration of the axial plane so that the sizes at different positions can be measured. If the simulation results are exact enough, then the container geometry should be in good agreement with the experimental one. Table 3 shows the container sizes from both the experimental and modeling results.

When compared with the experiment and simulation results, the size difference in diameter and height of the container fell below 5% (Table 3), indicating the accurate prediction of modeling.

The results clearly showed that HIP could effectively densify the specimen, and the relative density reached more than 99.7%. The overall size of the container decreased before and after hot isostatic pressing (HIP), and the middle part decreased the most, which is consistent with the result that the middle part of the specimen first starts to densify and then becomes densified. The DEFORM finite element simulation could accurately predict the container’s geometric change at different positions, and the difference between the predicted size and experimental one was less than 2%. It is noteworthy that the modeling results underestimated the axial deformation in the middle of the specimen. When the size was large within the container, the modeling error was correspondingly larger.

It is hypothesized that the rapid densification of the specimen at the beginning of HIP leads to a rapid change in the size of the container. However, the change in the whole capsule at this stage is not uniform, and the simulation process will idealize the entire densification process and continue this contraction, resulting in the simulated result being smaller in this area than the experimental result, yet the geometry of the HIPed part is roughly the same as the experimental measurement. Lastly, it should be noted that the filling density of powder will affect the densification rate and the relative density distribution during HIP [35].

## 5. Conclusions

The densification process of 316 L stainless steel powder using the hot isostatic pressing technique was modeled using finite element and validated by experimental results; the conclusions are as follows:

(1) The densification distribution of the HIPed billet was radial, and the densification rate of the central part was slower than that of the corner.
(2) The 316 L stainless steel powder could be densified with a relative density over 99% processed at 1050 °C/130 MPa/4 h, 1150 °C/130 MPa/4 h, and 1250 °C/130 MPa/4 h, respectively. The size difference between the simulation and experimental results of relative density of specimens for any position was less than 2%.
(3) Increasing the temperature could lead to faster densification.
(4) Experimental validations demonstrated that the model could accurately predict the changes in container size and relative density during HIP process.

## Figures and Tables

**Table 1:** The composition of 316 L stainless steel.

Element	C	Si	Mn	Mo	Ni	Cr	Fe
Wt.%	0.023	0.73	1.74	2.66	13.1	17.3	Bal.

**Table 2:** The composition of low-carbon steel (US SAE 1020) used to manufacture the container.

Element	C	Mn	P	S	Si	Cr	Ni	Cu	Fe
Wt.%	0.18	0.39	0.014	0.005	0.18	0.01	0.01	0.01	Bal.

**Table 3:** Container sizes in different positions based on the experiment and simulation.

Position	Experimental size (mm)		FEM dimension (mm)		Error (%)
	Before HIP	After HIP	Before HIP	After HIP	
①	73.4	68.66	73.4	67.53	−1.65
②	71.1	68.66	71.1	68.68	0.03
③	71.1	68.66	71.1	68.68	0.03
④	71.1	64.04	71.1	61.43	−4.08
⑤	71.1	64.20	71.1	65.24	1.62
⑥	71.1	64.20	71.1	65.24	1.62

## Data Availability

Data are contained within the article.
